# Isolation, Characterisation and Experimental Evolution of Phage that Infect the Horse Chestnut Tree Pathogen, *Pseudomonas syringae* pv. *aesculi*

**DOI:** 10.1007/s00284-020-01952-1

**Published:** 2020-03-19

**Authors:** Sarah L. James, Mojgan Rabiey, Benjamin W. Neuman, Glynn Percival, Robert W. Jackson

**Affiliations:** 1 School of Biological Sciences, University of Reading, Whiteknights, Reading, RG6 6AJ UK; 2 College of Arts, Sciences and Education, Biology Department, Texarkana, TX 75503 USA; 3 Bartletts Tree Experts, Shinfield, Reading, RG2 9DH UK

## Abstract

Bleeding canker of horse chestnut trees is a bacterial disease, caused by the bacterium *Pseudomonas syringae *pv.* aesculi,* estimated to be present in ~ 50% of UK horse chestnut trees. Currently, the disease has no cure and tree removal can be a common method of reducing inoculum and preventing spread. One potential method of control could be achieved using naturally occurring bacteriophages infective to the causative bacterium. Bacteriophages were isolated from symptomatic and asymptomatic horse chestnut trees in three locations in the South East of England. The phages were found to be belonging to both the *Myoviridae* and *Podoviridae* families by RAPD PCR and transmission electron microscopy. Experimental coevolution was carried out to understand the dynamics of bacterial resistance and phage infection and to determine whether new infective phage genotypes would emerge. The phages exhibited different coevolution patterns with their bacterial hosts across time. This approach could be used to generate novel phages for use in biocontrol cocktails in an effort to reduce the potential emergence of bacterial resistance.

**Electronic supplementary material:**

The online version of this article (10.1007/s00284-020-01952-1) contains supplementary material, which is available to authorized users.

## Introduction

In the last 15 years, there has been an epidemic outbreak of bleeding canker disease in horse chestnut tree populations in North Western Europe [1, 2]. Horse chestnut bleeding canker (HCBC) is a disease caused by the bacterium *Pseudomonas syringae *pv. *aesculi* (*Pae*). Disease symptoms are observed as dark lesions on the trunk and branches with tree exudates emerging from the lesions to create a bleed. The phloem, cambium and aerial woody parts of the tree are infected, and the leaves of the tree may become chlorotic and drop early [3]. If the canker infection becomes acute and encircles the entire trunk then the water supply can be cut off to the crown and the tree will die. Pockets of dead tissue caused by the cankers can lead to the trees developing weakened limbs and trunks, thus being deemed unsafe and removed due to the potential for collapse [4].

*Pseudomonas syringae* is a species complex of plant pathogens that can infect a wide range of plant hosts. The pathogen can be widely dispersed in the environment via its link to the water cycle [5, 6]. Two distinct pathotypes of *Pae* have been found, one (called *I-Pae*) that causes a leaf spot disease in *Aesculus indica* (Indian horse chestnut), and another (called *E-Pae*) that causes HCBC in *Aesculus hippocastanum* (European horse chestnut). Multilocus sequence typing using seven housekeeping genes found that the *E-Pae* strains were highly related to the *I-Pae* strain [7], but with just a few key genomic differences that likely reflects the difference in pathology. Genome sequencing of three British *Pae* strains displayed strong genomic similarity implicating a recent introduction of the pathogen into the country. Little is known about the route of transmission of *Pae* and how it can gain entry into the tree and survive within the tree environment. However, establishment of infection means the tree can rapidly succumb leading to death [3].

Tree disease has rapidly come to the forefront of national plant health agendas because of the threat of major population losses and the inability to treat trees with microbial infections [8]. One potential approach to consider is bacteriophage (phage) therapy of trees with bacterial infections [9]. Phage are viruses of bacteria, which can rapidly infect and kill bacteria without harming the host [10]. Phage have long been used for the control of human and plant bacterial diseases, but their usage is limited in many countries because of the prevalence of antibiotic and chemical treatments [9–11]. However, regulatory changes and the advent of widespread antibiotic resistance has brought a reappraisal of phage as an application for disease treatment. The development of effective phage biological control agents requires knowledge of the phage, the host bacterium and the way in which they interact. Studying how the two coevolve together can aid us in understanding the dynamics of bacterial resistance and phage infection [12] that will help with understanding the way in which they will work in the field and to the design of a targeted phage therapy agent or cocktails of phage [13, 14]. Here, we aimed to isolate, identify and characterise phage that can infect *Pae*. We then aimed to examine how the phage and host coevolved with a view to identifying patterns of evolution and new phage genotypes that could be used in kill curve assays to identify the efficacy of dual-phage cocktails for preventing host bacterium growth.

## Materials and Methods

### Bacterial Strains and Culture Media

*Pseudomonas syringae pv. aesculi* (*Pae*) strain 2250 was used as the host strain for isolation and propagation of phage isolates. Other *Pae* strains (*Pae* 6617, 6619, 6620, 6623, 6631) and bacteria were used for analysis of host range (Table 1). Kings B Medium (KB, 1 L distilled water, 20 g protease peptone, 1.5 g K_2_HPO_4_, 1.5 g MgSO_4_·7H_2_O, 10 ml glycerol (plus 15 g L^−1^ agar for plates); [15]) was used for culturing the bacterial strains. For broths, a single colony was inoculated into 10 ml of KB within 30 ml glass vials (ThermoFisher Scientific, UK) and incubated at 27 °C for 16 h in a shaking incubator (200 rpm). For agar plates, the bacteria were streaked on plates (48 h) or added as a suspension (10^8^ colony forming unit (CFU) per ml) in hard agar (1.5% agar) bottom plates (24 h) for plaque assays. 0.7% KB agar (diluted from 1.5% to 0.7% with sterile distilled water (SDW) in appropriate volumes for the number of assays required) was used to prepare the soft top agar overlay of the plaque assays. Glycerol (40% v/v with SDW) was used 1:1 with overnight bacterial broth suspensions for frozen stock production.

**Table 1:** Bacterial strains used in this study

Bacterium	Strain	Source
*Pseudomonas syringae *pv.* aesculi*	2250	Horse Chestnut in Pitlochry, UK, Green et al. [8]
*Pae*	6617	Horse Chestnut in Glasgow, UK, Green et al. [8]
*Pae*	6619	Horse Chestnut in Winchester, UK
*Pae*	6620	Horse Chestnut in Ewelme, UK
*Pae*	6623	Horse Chestnut in Alice Holt, UK
*Pae*	6631	Horse Chestnut in Belgium, Bultreys and Gheysen [1]
*P. syringae* pv. *corriandricola*	12,471	Yamamoto et al. (2000)
*P. syringae* pv. *lachrymans*	789	Cucurbit pathogen, Yamamoto et al. (2000)
*P. syringae* pv. *tomato*	DC3000	Tomato pathogen, Buell et al. (2003)
*P. agaricii*	NCPPB 2472	Mushroom pathogen, Storey (2018)
*P. cichorii*	907	Zannoni and Ingledew (1984)
*P. corrugata*	CFBP5454	Tomato pathogen, Solaiman et al. (2005)
*P. fluorescens*	Pf-01	Soil, Compeau et al. (1988)
*P. fluorescens*	Pf-5	Rhizosphere of cotton seedlings, Howell and Stipanovic (1979)
*P. putida*	PAW340	Soil, Franklin and Williams (1980)
*P. tolaasii*	2192T	Mushroom pathogen, Storey (2018)
*P. viridiflava*	ICMP 2848	Bean^a^
*P. marginalis *pv.* marginalis*	NCPPB 247	Lettuce^b^
*P. marginalis* pv. *pastinaceae*	NCPPB 949	Lettuce^c^
*Citrobacter werkmanii*	CwR94	Strawberry, Hamilton (2015)
*Erwinia amylovora*	CFBP 1430	*Crataegus* sp. pathogen, Paulin and Samson (1973)
*Rhizobium leguminosarum* bv *trifolii*	RCR221	White clover, Roberts et al. (2017)

*Pseudomonas syringae* pv. *aesculi* (*Pae*) 2250 was used as the host strain for isolation and propagation of phage isolates. Others strains of *Pae*, alongside other Pseudomonads and environmental bacteria, used for analysis of host range

^a^https://ncppb.fera.defra.gov.uk/furtherinfo.cfm?ncppb_no=247

^b^https://ncppb.fera.defra.gov.uk/furtherinfo.cfm?ncppb_no=949

^c^https://scd.landcareresearch.co.nz/Specimen/ICMP_2848

### Sample Collection from Trees

Samples were taken from ten *A. hippocastanum* trees showing symptoms of HCBC disease and ten asymptomatic trees, on the University of Reading Whiteknights Campus (Reading, UK). Other samples were taken from four diseased and four healthy trees at Kew Gardens (Royal Botanic Gardens, London, UK); and from one diseased tree and three healthy trees at Harpsden woods near Henley-on-Thames (Oxfordshire, UK). Soil samples were taken from the base of the tree, no more than a metre from the tree, from 10 cm into the ground and taking lower soil rather than surface soil. The trowel used to take the soil sample was sterilised with 100% ethanol between trees. Leaf samples were taken from the lower branches of the tree, with 3 whole leaflets being taken. All samples were stored at 4 °C for later use.

### Sample Preparation for Phage Isolation

For phage isolation from leaf samples, one leaf was cut up using scissors (ethanol sterilised and flamed) and then placed inside a sterile 30 ml vial; 10 ml of phosphate buffered saline (PBS, prepared according to manufacturer’s instructions, ThermoFisher Scientific, UK) was added in addition with 1 g sterile glass beads. The vial was vortexed for a minute and 2 ml of resulting supernatant was filtered through a 0.22 µm syringe injection filter (Millipore, Sigma-Aldrich, UK) to remove any bacteria. The resulting filtrate was stored at 4 °C for use in plaque assays. For phage isolation from soil samples, 1 g of soil was weighed and added to a sterile 30 ml glass vial and the same method as for leaf samples was followed. After vortexing, the soil was allowed to sediment to the bottom of the vial and 2 ml of supernatant was then removed without disturbing the settled soil and filtered as described above. Bark samples (1 g) were treated in the same way as soil.

### Host Range Assay

The optical density (OD) of overnight bacterial cultures were measured at 600 nm and adjusted to an optical density of 0.1 (≃10^8^ CFU per ml). 100 µl of the bacteria were spread plated on to a hard KB agar plate to form a fresh lawn of the bacteria. The lawn was left to dry before 5 µl of different phage isolates (10^4^–10^5^ PFU ml^−1^) were spotted on to the plates and allowed to dry in a laminar flow hood. Controls of PBS were spotted on to the bacterial plates. When the plates were dry, they were incubated at 27 °C for 24 h. After incubation, plates were checked for clearing where the spots of phage were applied.

### Electron Microscopy

10 µl of fresh phage sample (≃10^8^–10^9^ PFU ml^−1^) was centrifuged in an ultracentrifuge filter tube (30 K, ThermoFisher Scientific, UK) for 30 min at 15,000 rpm at 4 °C and applied to the shiny side of a copper coated carbon-formvar TEM grid and left for 10 min to dry to the grid. Excess liquid was blotted away with filter paper. 10 µl of 1% (w/v in distilled water) uranyl acetate was spotted on to the grid and left for 1 min. Excess uranyl acetate was blotted away with filter paper. The grids were left to dry for 5 min in a fume hood. Samples were analysed using a Phillips CM200 transmission electron microscope (TEM) at an accelerating voltage of 80 kV and photos taken using the Reading AMT camera system software.

### Phage DNA Extraction

A ‘Phage DNA Isolation Kit’ (Norgen Biotek, Canada) was used to extract phage DNA isolated from a high titer plate lysate (minimum of 10^8^ PFU ml^−1^). Before DNA extraction the lysate was centrifuged at 13,000 rpm for 1 min then filtered through a 0.22 µm filter (Millipore) to remove any whole bacterial cells. The sample was then treated with the DNase and DNase buffer (Norgen Biotek, Canada) according to manufacturer’s instructions, for 15 min at room temperature. All other additional steps were followed (except for addition of proteinase K) and a second elution of the DNA undertaken to increase the volume eluted to 150 µl. Extracted DNA was stored in a 1.5 ml ultracentrifuge tube at − 20 °C until needed. The DNA concentration was measured using a Nanodrop 2000 (ThermoFisher Scientific, UK) by pipetting 2 µl of DNA sample onto the pedestal.

### Random Amplification of Polymorphic DNA (RAPD) PCR

RAPD PCR was done using DNA Primer P1 (CCGCAGCCAA [16]) at a concentration of 100 pmol µl^−1^, with 10 µl GoTaq® Green Mastermix (Promega, UK) and 8 µl SDW and added to a 200 µl PCR microtube. *Pae* 2250 was used as a control alongside a negative control containing only SDW. Reaction conditions were followed as described by Gutierrez, Martin-Platero [17]: four cycles at 94 °C for 45 s, 30 °C for 120 s and 72 °C for 60 s; 26 cycles at 94 °C for 5 s, 36 °C for 30 s and 72 °C for 30 s (the extension step increased by 1 s for every new cycle); a final step of 10 min at 75 °C followed by 4 °C for ∞. DNA amplicons were electrophoresed through a 1% (w/v) agarose (Bioline, UK) gel and bands excised from the gel and purified with a QIAGEN clean-up kit (QIAGEN, UK) to be cloned for sequencing. DNA fragments were cloned using the TOPO One-shot system according to manufacturer’s instructions (Invitrogen, ThermoFisher Scientific, UK).

### Experimental Coevolution

30 ml glass vial containing 6 ml of KB broth were inoculated with 6 µl 3 × 10^7^ CFU ml^−1^ of *Pae* 2250 and 10 µl 10^3^ PFU ml^−1^ of phage without shaking [18] to hold the bacteria and phage population constant for varying number of transfers. Six replicate vials were set up for each different phage. Six control vials containing only KB broth was also used throughout the experiment to check for contamination. All the vials were incubated for 48 h at 27 °C after which the vials were vortexed and 60 µl of each population (including 60 µl of the control) was removed and transferred to a fresh glass vial containing 6 ml of KB broth. This was repeated once more after which a sample of the bacterial population and the phage population was removed and frozen at − 80 °C. Bacteria were isolated by spinning down 1 ml of the population, removing the supernatant and washing with 1 ml of PBS twice. 700 µl of bacterial suspension was mixed with 300 µl of 40% glycerol and frozen at − 80 °C. Phage were isolated by filtration of 1 ml of the population through a 0.22 µm filter. The experiment was continued for 12 transfers with sample population collections every 2nd transfer. Each phage was tested against the bacterial populations to past (two transfers previous), the present transfer (contemporary) and future (two transfers subsequent). Lines of each phage from each 2nd transfer were streaked on to large Petri dishes containing KB agar. Twenty colonies of each bacterium from each transfer were streaked across the phage line and the plates incubated for 48 h before observing for signs of bacterial infectivity. A bacterial colony was classed as sensitive to a phage population if there was any inhibition of growth, otherwise it was classed as resistant. The rate of phage infectivity evolution at each transfer was determined as proportion of resistant colonies of bacterial populations to past, contemporary and future of sympatric phage populations [19]. This calculation will provide a slope in which both phage infectivity evolution and bacterial resistance evolution can be determined.

### Killing Curve Assay

A killing curve assay was conducted to evaluate whether there were any differences in bacterial killing between past and future phage from the coevolution experiment. A Greiner 96-well flat-bottomed plate was inoculated with 100 μl of 10^8^ CFU ml^−1^ of overnight culture of bacterial cells in KB. Wells were then inoculated with 100 μl of 10^7^ PFU ml^−1^ (MOI of 0.1) of each respective phage sample (or cocktail of past and future, passage 12) to be tested. Directly after inoculation the plate was loaded into a Magellan plate reader running Tecan software, measuring the optical density at 595 nm every 20 min for 24 h, with incubation at 27 °C and shaking for 10 s before each reading.

## Results

### Phage Isolation

To isolate phage, samples were taken from both symptomatic and asymptomatic horse chestnut trees in the south east of England. The soil, leaves and bark of horse chestnut trees were sampled. It was hypothesised that infected trees would most likely have phage present due to the presence of the bacterial host. However, healthy trees were also included, to determine if there was any correlation between tree health and presence/absence or frequency of phage. A total of 32 trees were sampled from the south of England, 15 showing disease symptoms and 17 without symptoms. Bark was only sampled from 20 trees and not sampled thereafter because phage was not isolated from any of the initial samples. However, phages were isolated from the leaves of five (33%) and the soil of eight (53%) of the trees showing disease symptoms. Of these samples, two (13%) of them had phage present in both the soil and leaves of the sample. Phage were isolated from the leaves of five (29%) and the soil of four (24%) of the trees without disease symptoms. Of these samples, two (12%) of them had phage present both in the soil and leaves of the sample. There was no significant difference (ANOVA) in the mean PFU ml^−1^ of phage isolated from (i) leaves of canker positive (symptomatic) or negative (asymptomatic) trees (*F* = 1.01, df = 1, *p* = 0.32); or (ii) soil of canker positive (symptomatic) or canker negative (asymptomatic) trees (*F* = 0.24, df = 1, *p* = 0.63). A Fishers exact test showed that there was no significant difference for the number of trees (symptomatic or asymptomatic) with phage present on the leaves or in the soil.

### Phage Trait Analysis

To identify different phage isolates, a number of phenotypic traits were assessed, including plaque size (a measure of morphology) and host range (a measure of specificity). A total of 22 phage isolates were assessed. Plaque sizes of phage isolates from the tree samples varied from less than 1 mm to 3–4 mm in diameter. Plaques with diameters of less than 1 mm were all isolated from the leaf samples. Soil samples yielded phage isolates producing large clear plaques and small pinhole plaques. Some phage isolated from leaves produced hazy plaques, possibly indicating a lysogenic infection cycle and these were not studied further. To test the host range of the phage isolates, a range of different bacteria at OD 0.1 were tested for susceptibility; by spotting 5 µl of different phage isolates (10^4^–10^5^ PFU ml^−1^) on a lawn of bacteria and looked for clearing where the spots of phage were applied. All of the phage isolates could infect five other E-*Pae* strains and some were able to infect other pathovars of *P. syringae*, encompassing the entire spot (Table 2). However, the phage isolates were unable to infect other plant-associated bacteria, *P. fluorescens* SBW25, *Erwinia amylovora* strain EaCFBP430, and *Rhizobium leguminosarum* pv. *trifolii* (Table 2). Notably, differences in host range were observed when inoculated with other *P. syringe* pathovars or *P. marginalis* pv. *marginalis*.

**Table 2:** Host range analysis of twenty-two phage isolates from leaves (L) and soil (S) of *A*. *hippocastanum* trees

Sample	2250	6617	6619	6620	6623	6631	*P. s. lachrymans*	*P. s. tomato*	*P. m. marginalis*
RC1 L	+	+	+	+	+	+	−	+	−
RC10 L	+	+	+	+	+	+	+	+	−
RC5 L	+	+	+	+	+	+	+	−	−
RC3C L	+	+	+	+	+	+	+	+	−
RC7C L	+	+	+	+	+	+	+	+	−
RC8C L	+	+	+	+	+	+	+	−	−
RC9C L	+	+	+	+	+	+	+	−	+
RC9 S	+	+	+	+	+	+	−	−	−
RC5C S	+	+	+	+	+	+	−	−	−
**RC5C S**	** + **	** + **	** + **	** + **	** + **	** + **	−	** + **	** + **
**RC8C S**	** + **	** + **	** + **	** + **	** + **	** + **	−	−	−
4 K L	+	+	+	+	+	+	+	−	−
4CK L	+	+	+	+	+	+	+	+	−
**2 K S**	** + **	** + **	** + **	** + **	** + **	** + **	−	−	−
2 K S	+	+	+	+	+	+	+	+	−
4 K S	+	+	+	+	+	+	+	−	−
4 K S	+	+	+	+	+	+	+	+	−
1CK S	+	+	+	+	+	+	−	−	−
1CK S	+	+	+	+	+	+	−	−	−
**1CK S**	** + **	** + **	** + **	** + **	** + **	** + **	−	−	** + **
3CK S	+	+	+	+	+	+	−	−	−
3H S	+	+	+	+	+	+	+	−	−

Highlighted text (bold) denote phage selected and used for further in-depth characterisation, coevolution and trial. Most phages were able to infect *Pseudomonas syringae *pv.* aesculi* (2250*,* 6617*,* 6619*,* 6620*,* 6623, 6631) alongside other Pseudomonads (*P. syringae* pv. *lachrymans*, *P. syringae* pv. *tomato*, *P. marginalis* pv. *marginalis*)

### Characterisation of Four Candidate Phage

**Fig. 1:**
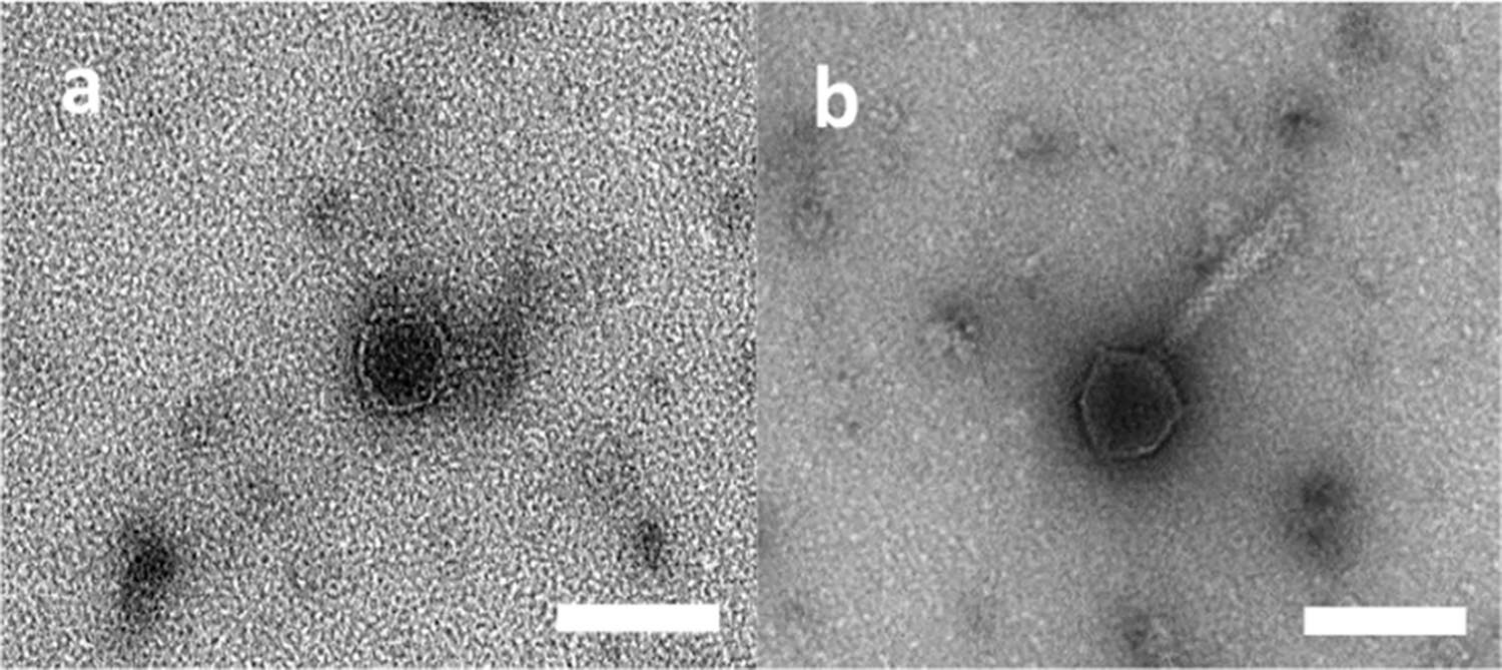
Morphology of two phages determined by transmission electron microscopy. **a** 2KS. **b** RC5CS. The size marker represents 50 nm

Four phage isolates, 2KS, RC8CS, RC5CS and 1CKS, were chosen for further analysis. Although they all came from soil samples, they were selected based on plaque size, with two of them being large plaque producing phage (2KS and RC8CS) and the other two producing small plaques (1CKS and RC5CS). These phage isolates also produced clear, not hazy, plaques indicating they were more likely to be lytic rather than lysogenic phage. All four exhibited a differential host range except 2KS and RC8CS. Transmission electron microscopy was employed to image the morphology of the phage and estimate their size (Fig. 1). All four phages exhibited typical head and tail morphologies associated with the Order *Caudovirales*. The two phages forming small plaques both displayed icosahedral heads of approximately 55 nm in size, a collar, and a long tail of roughly 90 nm and 120 nm in length for 1CKS and RC5CS, respectively. They had tubular tails with no visible tail fibres. Together, these characteristics suggest they belong to the *Myoviridae* family (Fig. 1). Although a clear image of phage RC8CS was difficult to obtain (Fig. 1d), both large plaque phage displayed icosahedral heads of roughly 50 nm in size, with a short tail with no tail fibres being obvious for 2KS (Fig. 1); these characteristics are typical of *Podoviridae* family phages.

### Molecular Characterisation

Unlike bacteria, there is no known conserved gene that is common to all phage. To gain further insight to the identity of the phage, Random Amplification of Polymorphic DNA (RAPD) PCR was employed to generate DNA fragments that could be cloned and sequenced. One amplified fragment (~ 4000 bp) was purified after electrophoresis and cloned and sequenced (Supplementary data Fig. 1). Although BLASTn analysis of the sequence did not return any matches for the four phage sequences, BLASTx returned matches. The two small myoviruses 1CKS and RC5CS exhibited 99% similarity to the phage phiPsa374. This phage belongs to the order *Caudovirales* and the family *Myoviridae* and infects *P. syringae pv. actinidae* (*Psa*), which causes disease in the kiwi fruit tree. Sequencing data also revealed the two small plaque *myoviridae* phages to have some protein sequence similarity to a PhoH protein. This PhoH protein could be found in several different phage as well as some bacteria and the protein has been used for identification of water borne bacteriophages of the family *Myoviridae*, further suggesting that the two phages are genetically similar and supporting their classification as belonging to the *Myoviridae* family. The sequences of the two large plaque phage 2KS and RC8CS revealed that both phages had similarities to the *Pseudomonas tolaasii* infecting bacteriophage Bf-7, which is a large plaque producing phage, belonging to the order *Caudovirales* and the family *Podoviridae*. Phage 2KS sequence also had similarities to a hypothetical protein in *E*. *coli* phage phiKT, which is a *podoviridae* phage. The phage RC8CS sequence also showed similarities to the capsid protein of phage Bf-7, suggesting the two could be classified into a similar group within the *Podoviridae* phage family and strengthens the argument for the classification of the phage RC8CS, whose TEM identification was tentative, as a member of the *Podoviridae*.

### *Killing Curves of the Four Phage Against* Pae

To examine the effects of each of the four phages on bacterial killing, a series of experiments were done to establish killing curves. All four phages inhibited bacterial growth although the emergence of bacterial growth likely associated with phage resistance was observed with all four phages. *Pae* 2250 growth increase happened quickest with phage RC8CS, then 2KS, and approximately equally for RC5CS and 1CKS, with an average emergence time of 16 h.

This led us to analyse the bacterium-phage co-evolutionary relationship to determine whether new infective phage genotypes would emerge and to understand the co-evolutionary dynamics.

### Patterns of Co-evolutionary Dynamics

To examine the patterns of coevolution between the four phages and *Pae* 2250, the bacterium and the four different phages were grown in glass vial microcosms containing KB medium. A proportion of the coevolving population was transferred to a fresh microcosm every 2 days and this was continued for 12 transfers. The proportion of bacteria that had developed resistance to the phage was determined by phage-bacteria streak plates to look for “breaks” (loss of growth) where the bacterial line crossed the phage line. This process was repeated for all 6 time transfer points and using either past, contemporary or future phage for each time transfer point.

**Fig. 2:**
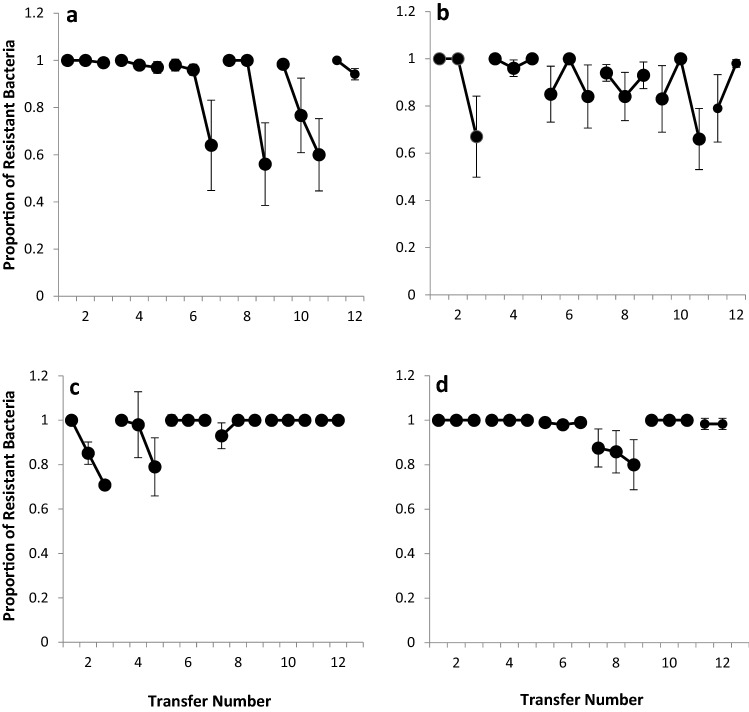
Rate of evolution of phages through time. **a** RC8CS, **b** 2KS, **c** 1CKS, **d** RC5CS. The experimental coevolution was done by inoculating King B medium (KB) with phage and bacteria. After 48 h incubation at 27 °C, both bacteria and phage were recovered and transferred to new KB broths. This was repeated for 12 transfers with sample population collections every 2nd transfer. Each line represents the resistance of bacteria to past (two transfers previous), contemporary (the present transfer) and future (two transfers subsequent) phage. Values are mean of six replicates ± SEM

The bacterial resistance to the past RC8CS phage was high throughout the experiment even after the 2nd transfer, being > 98% across all transfer numbers (Fig. 2). The proportion of bacteria resistant to the future phage rapidly decreases at the 6th transfer with the proportion not being any higher than 64%. The negatively sloping line observed from transfer 6 indicated an escalatory evolution of phage infectivity in the population. This pattern of coevolution fits with that observed in arms race dynamics where allele mutations increase in frequency over time. A different pattern of evolution across time was observed for the other large plaque phage 2KS with no evidence of a culmination of escalatory evolution of resistance. Instead, an oscillating pattern was observed with bacteria either being more resistant to their contemporary phage than the past or future; or being more susceptible to their contemporary than the past or future.

There was initially evidence in the first two transfers of phage 1CKS that the phage was coevolving with its host depicting arms race dynamics with escalating levels of susceptibility. However, from transfer 6 the bacterial population at each transfer shows a high level of resistance to their past, contemporary and future phage with resistance > 80% for all tests, with exception for transfer 8, which showed a small decrease in resistance to the past phage. For the other small plaque phage, RC5CS, bacterial resistance to the phage was > 98% across all transfers except transfer 8 where there was a drop in total resistance to 80%; however, the line still remained flat indicating no level of escalatory evolution.

The proportion of resistant bacteria across all phage tests and transfers never fell below 66%. Statistical analysis of the four phages using a repeated measures model, showed that there was a significant difference over the course of the experiment for all 4 phage including the time shift (*p* = 0.001). There was a significant difference between the phage over time (*p* = 0.003) and between phage and the time shift (*p* = 0.004).

Finally, to see if there were phage still present in the final time point populations from the coevolution experiment, 5 µl of the phage population was spot plated on to a lawn of past bacteria. All six replicates for each individual phage lysed the bacterial lawn encompassing the entire spot. This indicates that the phage population did not go extinct even though the observations may have suggested this due to the high resistance of the evolved bacterial population.

### Some Evolved Phage Provide More Durable Control of Bacterial Growth

**Fig. 3:**
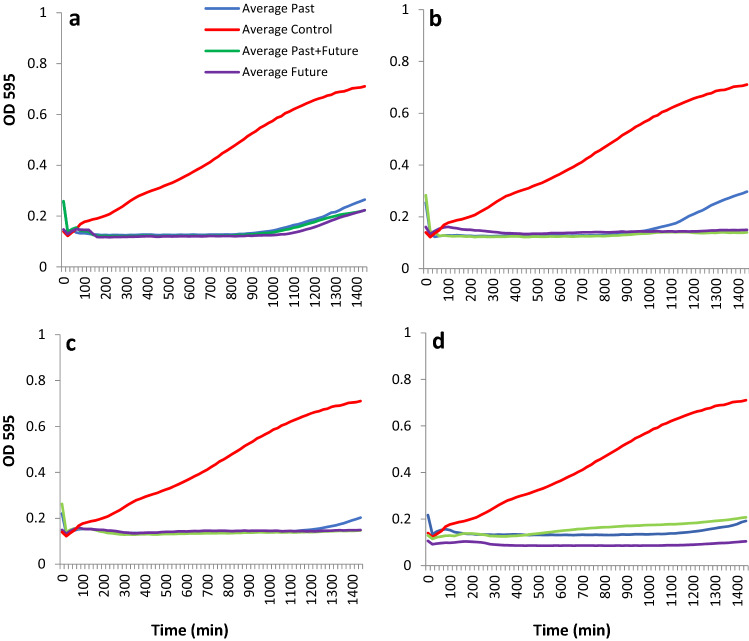
In vitro killing curve of past and future phages. **a** RC8CS, **b** 2KS, **c** RC5CS, **d** 1CKS phage on the bacterium *Pseudomonas syringae *pv. *aesculi* 2250 (control) using MOI of 0.1. Past, wildtype phage and future phage isolated from the coevolution end point (passage 12). Past: phage collected and tested from two transfers previous and future: phage collected and tested from two transfers subsequent to present phage. The plate was incubated at 27 °C and the OD_595_ was measured every 20 min for 24 h with shaking for 10 s before each reading. Values are the means of 6 replicates

The evolution of new phage genotypes provided an opportunity to test them out both individually and in combination with its cognate parent for inhibition of bacterial growth, especially where resistant bacteria emerged. Only the 2KS and RC5CS evolved phage (singly or in combination with their cognate wildtype) exhibited the ability to inhibit bacterial growth beyond the time when bacterial growth emerged for the wildtype phage alone (Fig. 3).

## Discussion

Bleeding canker of horse chestnut trees poses a great threat to the species in the UK and the rest of Europe, yet no control strategy has been found. Without appropriate intervention we may face the loss of an amenity species. To identify novel control strategies requires considerations of new therapies, especially since antibiotic usage is restricted. Phage therapy offers the opportunity to develop and use a natural biocontrol agent. Therefore, in this research, we identified and characterised phage and through the use of experimental evolution obtained new phages. A number of bacteriophages from diseased and non-diseased tree materials were isolated and tested for infectivity against *Pae*, the causative agent of bleeding canker in horse chestnut. The bacteriophages inability to infect *P. fluorescens* and *R. leguminosarum* pv. *trifolli* is promising for phage treatment, as these are both plant beneficial bacteria: *P. fluorescens* plays a role in plant growth promotion [20] and *Rhizobia* are important in the nitrogen cycle and improving nitrogen availability to plants. Therefore, we would not want to negatively impact such beneficial bacteria with any plant phage-based biocontrol agent. Equally, it is very positive that the phages are able to infect all the isolates of *E-Pae*, as this indicates that it would be able to infect multiple strains in the field, especially given the *Pae* strains exhibit a high degree of similarity [7]. Notably, *E-Pae* strain (6631) comes from Belgium. However, the host range assay in this study was done using spot tests which might overestimate both the overall virulence and the host range of phage and future testing should consider quantitative approaches for accurate host range determination [22, 23].

The phage isolates were coevolved with the host to create new genotypes, enabling exposure bacterial defence mechanisms and thus favouring the emergence of new phage genotypes that can overcome the bacterial defences. Doing this over several passages allowed insight to the phages abilities to coevolve with its host and the implication such adaptation may have upon fitness [12, 24]. The coevolved phages might demonstrate greater ability to combat bacterial resistance. The experimental coevolution allowed insight to whether evolution of bacterial resistance was escalatory i.e. the future phage being more infective than the contemporary and the contemporary being more infective than the past, indicated by a negative sloping line. The experiment showed the two small plaque *Myoviridae* phages were more limited in evolutionary innovations during coevolution with their host. However, a different observation was made for the large plaque producing *Podoviridae* phages, with each displaying different patterns of coevolution. Previous studies using *Podoviridae* phages have demonstrated coevolution within the population [26]. This would suggest that identification of *Podoviridae* phage specific to the bacterium of interest could be beneficial in the creation of a phage therapy. The pattern in *Podoviridae* phage 2KS is an oscillating pattern of peaks and troughs across time, commonly seen in the matching alleles pattern of coevolution [27] but this pattern can also indicate an arms race under fluctuating selection dynamics. However, the dynamics for the other *Podoviridae* phage RC8CS shows negative slopes of resistance across time, the pattern seen in the gene-for-gene pattern of coevolution. An alternative explanation may be the presence of a mutator strain of the bacterium present in the population giving rise to differences between the two, although the presence of mutators within a population is likely to send the phage population extinct [28], something not observed in this coevolution study. This pattern of evolution has similarities to that observed in fluctuating selection dynamics, either following matching alleles-type dynamics or the result of ecological feedback in arms race dynamics, although without comparative genomic information it is difficult to conclusively favour one over the other. We showed that experimental coevolution can be used as means for the generation of new phages better able to control the pathogen populations and thus can be considered for use in cocktails for biological control of plant diseases.

The results indicate the interaction between phages and bacteria is complex and is not the same across all phage and bacteria. However, this work provides insight for considering an approach to the application of phage as a tool for the treatment of plant disease, as it both encompasses insights in to dynamics and usage for bacterial control. The results show promise for future studies and design of phage control agents.

## Electronic supplementary material

Below is the link to the electronic supplementary material.

Supplementary file1 (DOCX 188 kb)

## References

[1] Bultreys A, Gheysen I, Fatmi MB, Collmer A, Iacobellis NS, Mansfield JW, Murillo J, Schaad NW (2008). Siderophore uses in *Pseudomonas syringae* identification. *Pseudomonas syringae* pathovars and related pathogens—identification, epidemiology and genomics.

[2] Schmidt O, Dujesiefken D, Stobbe H, Moreth U, Kehr R, Schroder T (2008). *Pseudomonas syringae* pv. *aesculi* associated with horse chestnut bleeding canker in Germany. For Pathol.

[3] Steele H, Laue BE, MacAskill GA, Hendry SJ, Green S (2010). Analysis of the natural infection of European horse chestnut (Aesculus hippocastanum) by *Pseudomonas syringae* pv. *aesculi*. Plant Pathol.

[4] Green C, Laue B, Fossdal CJ, A’Hara SW, Cottrell JE (2009). Infection of horse chestnut (*Aesculus hippocastanum*) by *Pseudomonas syringae* pv. *aesculi* and its detection by quantitative real-time PCR. Plant Pathol.

[5] Abramovitch RB, Kim YJ, Chen SR, Dickman MB, Martin GB (2003). Pseudomonas type III effector AvrPtoB induces plant disease susceptibility by inhibition of host programmed cell death. EMBO J.

[6] Block A, Alfano JR (2011). Plant targets for *Pseudomonas syringae* type III effectors: virulence targets or guarded decoys?. Curr Opin Microbiol.

[7] Maki LR, Galyan EL, Chang-Chien MM, Caldwell DR (1974). Ice nucleation induced by pseudomonas syringae. Appl Microbiol.

[8] Green S, Studholme DJ, Laue BE, Dorati F, Lovell H, Arnold D (2010). Comparative genome analysis provides insights into the evolution and adaptation of *Pseudomonas syringae* pv. *aesculi* on *Aesculus hippocastanum*. PLoS ONE.

[9] D’Herelle MF (1917). On an invisible microbe antagonistic to dysentery bacilli. C R Acad Sci.

[10] Jones JB, Jackson LE, Balogh B, Obradovic A, Iriarte FB, Momol MT (2007). Bacteriophages for plant disease control. Annu Rev Phytopathol.

[11] Marinelli LJ, Fitz-Gibbon S, Hayes C, Bowman C, Inkeles M, Loncaric A (2012). Propionibacterium acnes bacteriophages display limited genetic diversity and broad killing activity against bacterial skin isolates. Mbio.

[12] Brockhurst MA, Morgan AD, Rainey PB, Buckling A (2003). Population mixing accelerates coevolution. Ecol Lett.

[13] Carlton RM (1999). Phage therapy: past history and future prospects. Arch Immunol Ther Exp (Warsz).

[14] Lee WJ, Billington C, Hudson JA, Heinemann JA (2011). Isolation and characterization of phages infecting *Bacillus cereus*. Lett Appl Microbiol.

[15] King EO, Ward MK, Raney DE (1954). Two simple media for the demonstration of pyocyanin and fluorescin. J Lab Clin Med.

[16] Jaiswal A, Koley H, Ghosh A, Palit A, Sarkar B (2013). Efficacy of cocktail phage therapy in treating *Vibrio cholerae* infection in rabbit model. Microbes Infect.

[17] Gutierrez D, Martin-Platero AM, Rodriguez A, Martinez-Bueno M, Garcia P, Martinez B (2011). Typing of bacteriophages by randomly amplified polymorphic DNA (RAPD)-PCR to assess genetic diversity. FEMS Microbiol Lett.

[18] Morgan AD, Buckling A (2006). Relative number of generations of hosts and parasites does not influence parasite local adaptation in coevolving populations of bacteria and phages. J Evol Biol.

[19] Brockhurst MA, Morgan AD, Fenton A, Buckling A (2007). Experimental coevolution with bacteria and phage. The *Pseudomonas fluorescens*—Phi2 model system. Infect Genet Evol.

[20] Kloepper JW, Leong J, Teintze M, Schroth MN (1980). Enhanced plant growth by siderophores produced by plant growth-promoting rhizobacteria. Nature.

[21] Koskella B, Thompson JN, Preston GM, Buckling A (2011). Local biotic environment shapes the spatial scale of bacteriophage adaptation to bacteria. Am Nat.

[22] Gayder S, Parcey M, Castle AJ, Svircev AM (2019). Host range of bacteriophages against a world-wide collection of *Erwinia amylovora* determined using a quantitative PCR assay. Viruses.

[23] Miransari M (2010). Contribution of arbuscular mycorrhizal symbiosis to plant growth under different types of soil stress. Plant Biol.

[24] Gomez P, Buckling A (2011). Bacteria-phage antagonistic coevolution in soil. Science.

[25] Hall AR, De Vos D, Friman VP, Pirnay JP, Buckling A (2012). Effects of sequential and simultaneous applications of bacteriophages on populations of *Pseudomonas aeruginosa* in vitro and in wax moth larvae. Appl Environ Microbiol.

[26] Sorek R, Kunin V, Hugenholtz P (2008). CRISPR-a widespread system that provides acquired resistance against phages in bacteria and archaea. Nat Rev Microbiol.

[27] Scanlan PD, Buckling A (2012). Co-evolution with lytic phage selects for the mucoid phenotype of *Pseudomonas fluorescens* SBW25. ISME J.

[28] Abedon ST, Yin J (2009). Bacteriophage plaques: theory and analysis. Methods Mol Biol.

