# Supplementary material for: Isolation, Characterisation and Experimental Evolution of Phage that Infect the Horse Chestnut Tree Pathogen, Pseudomonas syringae pv. aesculi
Source: Curr Microbiol. 2020 Mar 19;77(8):1438–47. doi: 10.1007/s00284-020-01952-1 (PMC7334240; doi:10.1007/s00284-020-01952-1)
Supplement: Supplementary file 1 — Supplementary file1 (DOCX 188 kb) [file 284_2020_1952_MOESM1_ESM.docx]

**Supplementary data *for Current Microbiolog***

**Isolation, characterisation and experimental evolution of phage that infect the Horse Chestnut tree pathogen, *Pseudomonas syringae* pv. *aesculi***

Sarah L. James^1^, Mojgan Rabiey^1*^, Benjamin W. Neuman^2^, Glynn Percival^3^, and Robert W. Jackson^1^

^1^School of Biological Sciences, University of Reading, Whiteknights, Reading, RG6 6AJ, UK

^2^College of Arts, Sciences and Education, Biology Department, Texarkana, TX 75503, USA

^3^Bartletts Tree Experts, Shinfield, Reading, RG2 9DH, UK

^*^Corresponding author: Mojgan Rabiey, [m.rabiey@reading.ac.uk](mailto:m.rabiey@reading.ac.uk),

Tel: +44 (0) 1183787084


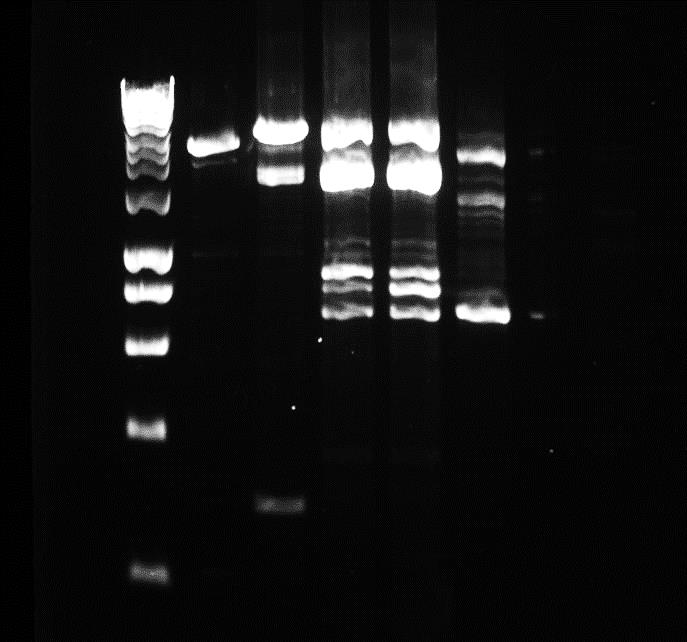


**1 2 3 4 5 6**

**Figure 1. RAPD Electrophoresis gel of the four characterised phage.** Lane 1 Hyperladder 1, Lane 2. RC8CS, Lane 3. 2KS, Lane 4. 1CKS, Lane 5. RC5CS, Lane 6. *Pae*
